# Professional Custom and Professional Caste

**Published:** 1898-12-31

**Authors:** 


### PROFESSIONAL CUSTOM AND PROFESSIONAL CASTE.

Professional customs are the growth of ages, and often but little except that fact can be said in their defence, and among them perhaps the least edifying is this cast-iron rule as to uniformity of fees. We make bold to say that there is not a junior in the Harley Street district who would not feel that he would be throwing away his chance of professional promotion were he to take a five-shilling fee, although he may sit whole mornings without seeing a single patient. Meanwhile, such of the seniors as have been lucky rake the money in, not, however, in most cases by dint of raising their fees, but by dint of multiplying their patients, seeing them by aid of double consulting-rooms, and so on, picking up a couple of guineas in one room while the last patient is buttoning her bodice in the other! This is not as it should be. If, when practice grows, men would raise their consultation fees and so ensure for themselves a reasonable rest, for their patients a more complete investigation, and for their juniors a larger practice, and if the juniors were allowed to accept openly and without loss of caste a junior's fee there would be no temptation to invent new plans or "consulting institutions" such as we have lately heard about.

